# A microfluidic approach to rescue ALS motor neuron degeneration using rapamycin

**DOI:** 10.1038/s41598-021-97405-1

**Published:** 2021-09-13

**Authors:** Phaneendra Chennampally, Ambreen Sayed-Zahid, Prabakaran Soundararajan, Jocelyn Sharp, Gregory A. Cox, Scott D. Collins, Rosemary L. Smith

**Affiliations:** 1 Microinstruments and Systems Laboratory, University of Maine, Orono, ME 04469 USA; 2 Graduate School of Biomedical Sciences and Engineering, University of Maine, Orono, ME 04469 USA; 3 The Jackson Laboratory, Bar Harbor, ME 04609 USA; 4 Department of Chemistry, University of Maine, Orono, ME 04469 USA; 5 Department of Electrical and Computer Engineering, University of Maine, Orono, ME 04469 USA

**Keywords:** Neuroscience, Engineering, Biomedical engineering

## Abstract

TAR DNA-binding protein-43 (TDP-43) is known to accumulate in ubiquitinated inclusions of amyotrophic lateral sclerosis affected motor neurons, resulting in motor neuron degeneration, loss of motor functions, and eventually death. Rapamycin, an mTOR inhibitor and a commonly used immunosuppressive drug, has been shown to increase the survivability of Amyotrophic Lateral Sclerosis (ALS) affected motor neurons. Here we present a transgenic, TDP-43-A315T, mouse model expressing an ALS phenotype and demonstrate the presence of ubiquitinated cytoplasmic TDP-43 aggregates with > 80% cell death by 28 days post differentiation in vitro. Embryonic stem cells from this mouse model were used to study the onset, progression, and therapeutic remediation of TDP-43 aggregates using a novel microfluidic rapamycin concentration gradient generator. Results using a microfluidic device show that ALS affected motor neuron survival can be increased by 40.44% in a rapamycin dosage range between 0.4-1.0 µM.

## Introduction

Motor Neurons (MNs) are specialized excitable cells whose somas reside strictly within the motor cortex or spinal cord. These neurons develop axons projecting to other MNs or remote peripheral effector organs, most notably muscles, and glands. As such, MNs carry a significant responsibility for nominal organismal control and function. Amyotrophic lateral sclerosis (ALS) is a singularly debilitating MN disease with an onset at either a single or multiple sites (limbs, bulbar or upper motor cortex) and progresses over time to other sites causing degeneration of both upper and lower motor neurons eventually leading to muscle atrophy and death^1^.

Superoxide dismutase 1 (SOD1), a protein misfolding^2^ enzyme, is one of the earliest definitive ALS genes identified in 1993^3^ and along with TAR DNA-binding protein-43 (TDP-43) aggregate formation in the cytoplasm^4^ are a common defining hallmark of ALS in both familial and sporadic pathologies. Currently, about 217 variants from point mutations spanning the SOD1 amino acid sequence have been identified in relation to ALS^5^ (ALSoD).

The mechanisms of TDP-43 aggregate formation in ALS are well documented^6^. Aggregate formation varies from patient to patient but is associated with abnormal C-terminal TDP-43 fragment^7,8^ or post-translational protein modification such as ubiquinitation^9,10^, phosophorylation^11,12^ and/or acetylation^13,14^ of the TDP-43 protein, resulting in disruption/mutation of the nuclear import signal of TDP-43^15,16^. It is commonly accepted that ALS mediates TDP-43 depletion from the nucleus with a concurrent accumulation of ubiquitinated cytoplasmic inclusions^17^, resulting in a loss of TDP-43 function as a nuclear protein as well as concurrent cytoplasmic toxicity resulting from mislocated TDP-43^18–20^. Recent studies suggest that TDP-43 mediated toxicity^21^ proceeds through recruitment and agglutination of RNA-binding proteins along with components of the ubiquitin–proteasome and autophagy-lysosome systems^22^. Another DNA binding protein mislocalization associated with familial ALS pathology is Fused In Sarcoma (FUS)^23,24^. FUS, a nuclear protein, has been associated with transcription, DNA repair processes^25,26^ and RNA metabolism^27^. However, the definitive reason for the mislocalization of the FUS protein is still inconclusive. Overall, mislocalized cytoplasmic TDP-43^28^ and FUS^29^ can be used as pathological disease markers of ALS.

A transgenic ALS mouse model with TARDBP mutation (TDP-43 A315T) was used in the current study. Although the use of approved human ES cell lines is becoming increasingly popular, a murine model was adopted here and carries significant advantages such as considerable in vitro data for experimental designs, ease of available markers and similar development pathway to that of humans. Though murine cell line has above listed advantages, it also has few limitations such as transgene overexpression or upregulation^30^ in mouse compared to human models and failure of pharmaceutical drugs^31^ in clinical trials though being successful in mouse models.

Rapamycin, or sirolimus, has been suggested as a potential therapeutic remediation for ALS^32,33^, and is widely used to inhibit mTOR signaling (the mechanistic target for rapamycin). The mTOR intracellular protein kinase complex is involved in a host of critical cellular processes, including protein synthesis, lipid synthesis, proliferation, and autophagy^34–36^. Autophagy is an essential cellular housekeeping function in which misfolded proteins and dead organelles are cleared from the cell. The mTOR complex, particularly the mTORC1 complex, inhibits autophagy through autophagy initiating kinase (ULK1) and ancillary downstream targets^37^. Rapamycin inhibits mTOR/mTORC1 signaling pathways^38^ subsequently activating autophagy^39^ and promoting abnormal protein clearing, including TDP-43 aggregates. Rapamycin inhibition of the mTOR pathways also activates lysosomal biogenesis^40^. Mandrioli et al. noted that rapamycin combined with riluzole, a glutamate blocker, can clear the cytoplasmic TDP-43 aggregates through induced autophagy^32^. In support of this, Kamm et al.^41^, using a microfluidic platform, reported that rapamycin produced increased muscle contractions in ALS derived motor units suggesting rapamycin as a potential ALS therapeutic.

Microfluidics has recently surfaced as an invaluable technique for cell culturing. One of the more significant advancements in microfluidics came with the advent of the concentration gradient generator, originally proposed by Whitesides^42^ and significantly refined by Demers^43,44^. Not only do gradient generators provide more facile, realistic, and meaningful bio environments for cellular studies, they can also provide custom spatial and temporal landscapes mimicking complex in vivo environments. This is a unique advantage of microfluidic systems that is not easily available using traditional cell plating techniques and therefore they provide a meaningful answers to previously intractable biological questions^43,45^. Microfluidics has since been incorporated successfully in many cell culture applications, including single-cell analysis, developmental studies, pharmacologic interventions, and precision medicine^46–48^. Here we recruit microfluidics to generate a linear rapamycin concentration gradient across a 3D MN culture and map the permissive zones of MN survival to evaluate the efficacy of rapamycin therapeutics in ALS. The results are compared with traditional 2D MN cultures.

## Results & discussion

ALS is clinically characterized by a general MN degeneration resulting in axonal retraction and pruning, eventually causing muscle atrophy and death. Cytoplasmic mislocalization of TDP-43 and FUS aggregates have been identified as biomarkers in familial ALS^49^ and are convenient metrics to investigate ALS pathology in MN cultures. In this study, a transgenic mouse model carrying the TDP-43-A315T (TARDBP) ALS phenotype was derived by crossing B6.Cg-Tg (Prnp-TARDBP*A315T) 95Balo/J mice with B6.Cg-Tg (Hlxb9-GFP)1Tmj/J mice (The Jackson Laboratories, JAX).The ES cells derived from this mutant line were differentiated into MNs in vitro and used to test the efficacy of rapamycin in rescuing the ALS phenotype.

### Characterization of motor neurons for ALS pathology

**Figure 1:**
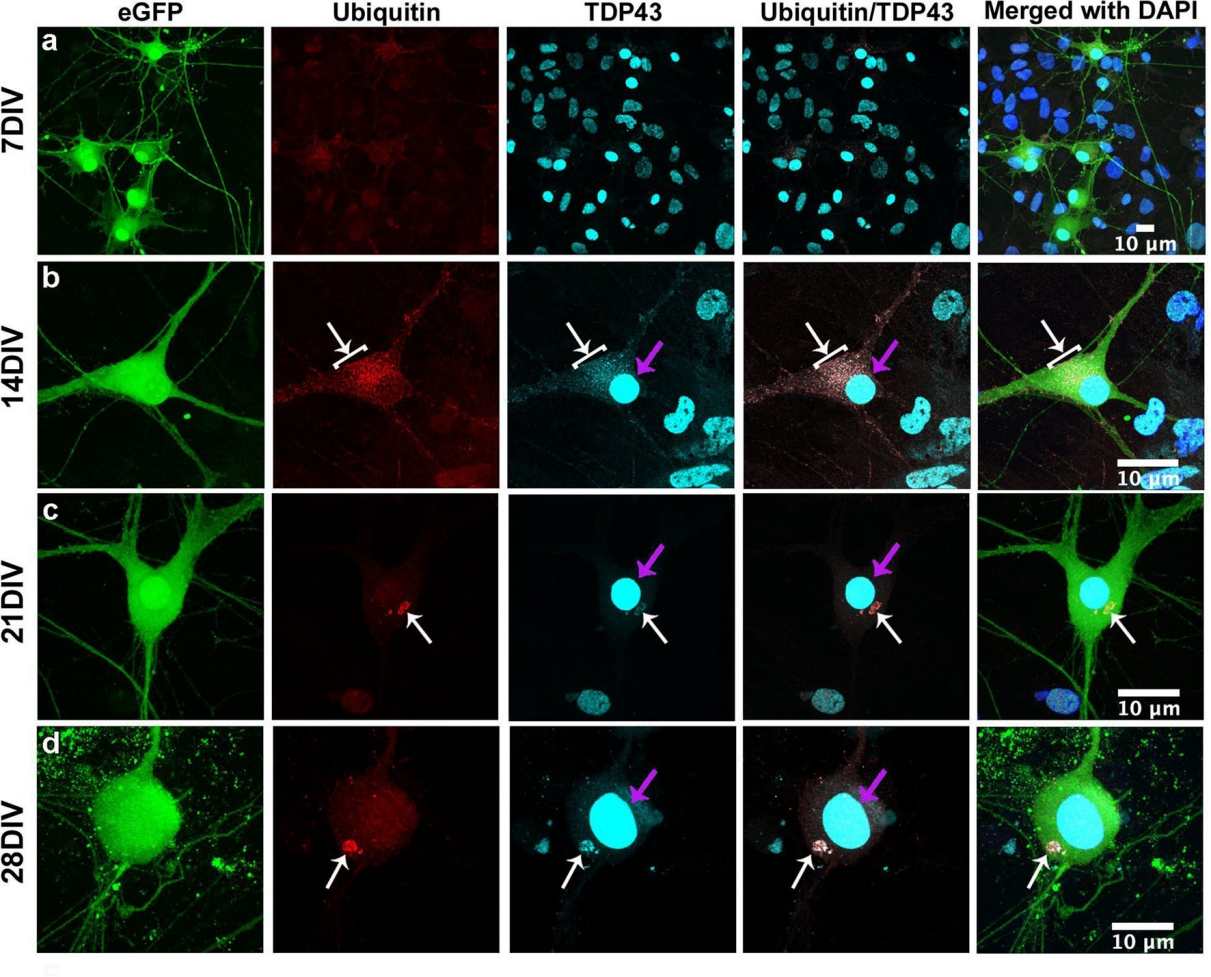
Immunocytochemical analysis of mutant MNs. Mutant MNs show the presence of both nuclear TDP-43 (pink arrows) and co-localized cytoplasmic TDP-43/ubiquitin aggregates (white arrows) at 7,14, 21 and 28 DIV (**a**–**d**). Massive dendritic pruning and axonal retraction in mutant MNs were observed at 28 DIV (**d**), indicating the onset of MNs degeneration. No disease phenotype was manifested at 7DIV in mutant cells (**a**). The MN nuclei can be identified from the large regions of TDP-43 staining.

**Figure 2:**
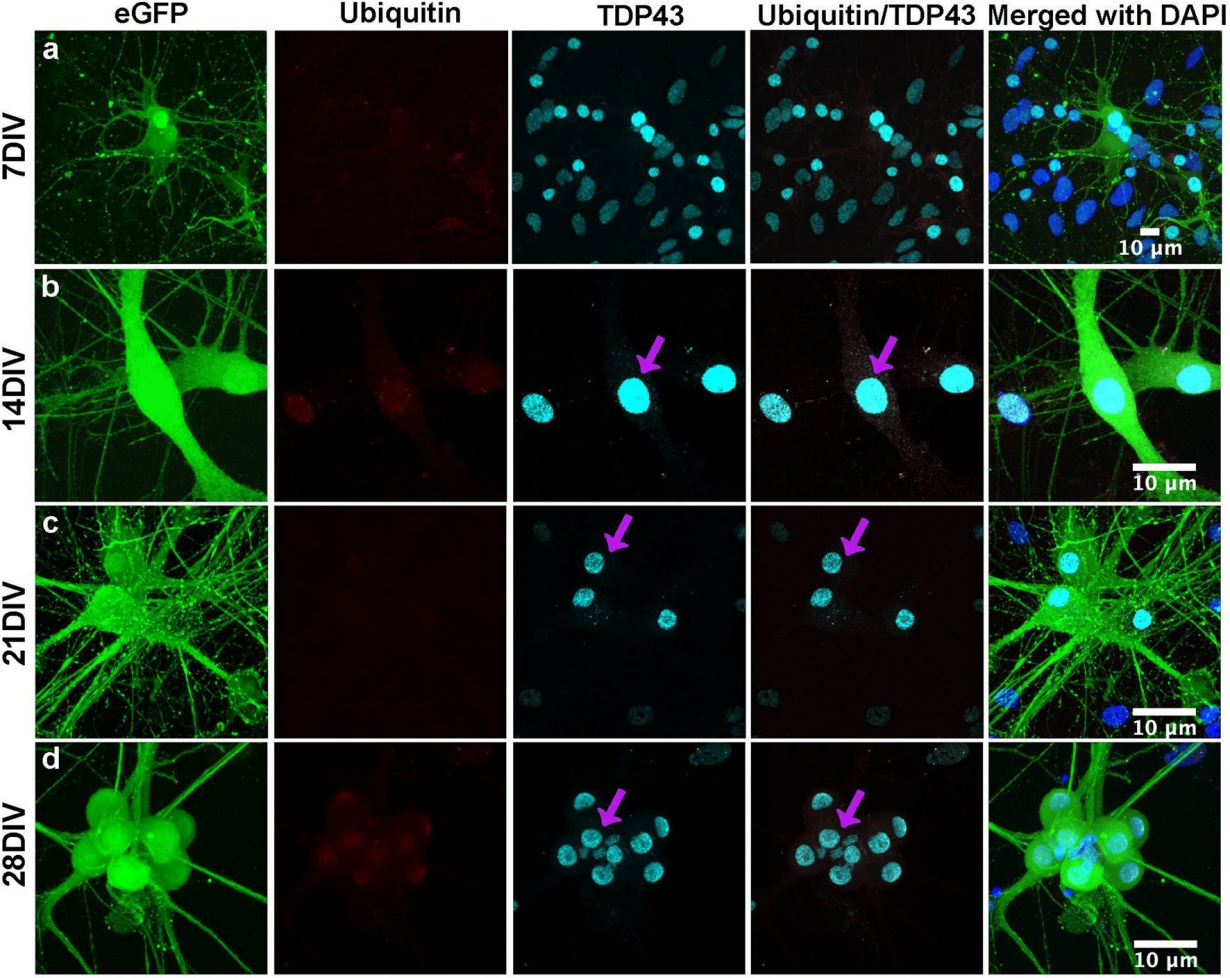
Immunocytochemical analysis of control MNs. Control MNs show the presence of only nuclear TDP-43 aggregates (arrows) and without any cytoplasmic aggregates of TDP-43/ubiquitin at 7DIV (**a**), 14DIV (**b**), 21DIV (**c**), and 28DIV (**d**). No disease phenotype was manifested, with healthy axons extension even at the end of 28DIV.

All MNs were cultured from embryonic stem cells (ES cells) containing an enhanced green fluorescent protein (eGFP) marker that is expressed under the control of the Hb9::GFP reporter gene to unequivocally identify differentiated MNs. ES cells containing only the GFP marker with no ALS mutation were cultured as control cells. Manifestation of an ALS phenotype in the TDP-43-A315T (TARDBP) mutant cell line was demonstrated by assaying the mutant MNs for TDP-43 cytosolic mislocalization. Stem cells were differentiated into MNs in a 2D culture and immunocytochemically stained for TDP-43 and ubiquitin at 7 days, 14 days , 21 days and 28 days in vitro (DIV) (Fig. 1a–d). As expected, nuclear TDP-43 expression was found in both mutant MNs (Fig. 1) and control MNs (Fig. 2) throughout development, while cytoplasmic TDP-43 aggregates appeared only in the mutant strain and only after 14 DIV (arrows; Fig. 1b–d). Ubiquitin showed diffuse expression for both mutant and control MNs, but was over expressed in mutant MNs at regions near, but outside the nucleus. These regions of over expressed cytosolic ubiquitin (arrows) were co-localized with cytosolic TDP-43 aggregates indicating possible ubiquitination of TDP-43 resulting in the formation of pathological TDP-43 aggregates.

It appears that all cultures demonstrated healthy development at initial MN stages, i.e. without any cytoplasmic TDP-43 agrregates, but mutant cell line progressed to a disease phenotype post 7DIV. Interestingly, the degree of nuclear TDP-43 expression in mutant and control MNs was similar with no sign of nuclear clearance, indicating a dependable role in carrying out transcriptional regulation and other nuclear functions^50^ despite the mislocalization of TDP-43. This is consistent with a recent study by Burkhardt et al.^51^ where human induced pluripotent stem cell-derived motor neurons carrying the TDP-43-A315T mutation maintained nuclear expression of TDP-43 while still demonstrating an ALS disease phenotype.

At 28 DIV we observed increased dendritic pruning and axonal retraction^52,53^ of mutant MNs (Compare Fig. 1d with Fig. 2d). There was also a correspondingly sharp increase in MN mortality for mutant MNs with only an 8.7 ± 5.7% survival at 28 DIV as compared to control MNs, 58.5 ± 16.2% survival at 28 DIV (*p* ≤ *0.001, n* ≥ *3*, with a two-way ANOVA-sidak comparison) (Fig. 3a). Additionally, control ES cell-derived motor neurons retained normal cell morphology, dendritic projections and nuclear TDP-43 expression (Fig. 2a–d) with complete absence of cytoplasmic TDP-43 or ubiquitin^+^ aggregates (Figure S5).

**﻿Figure 3:**
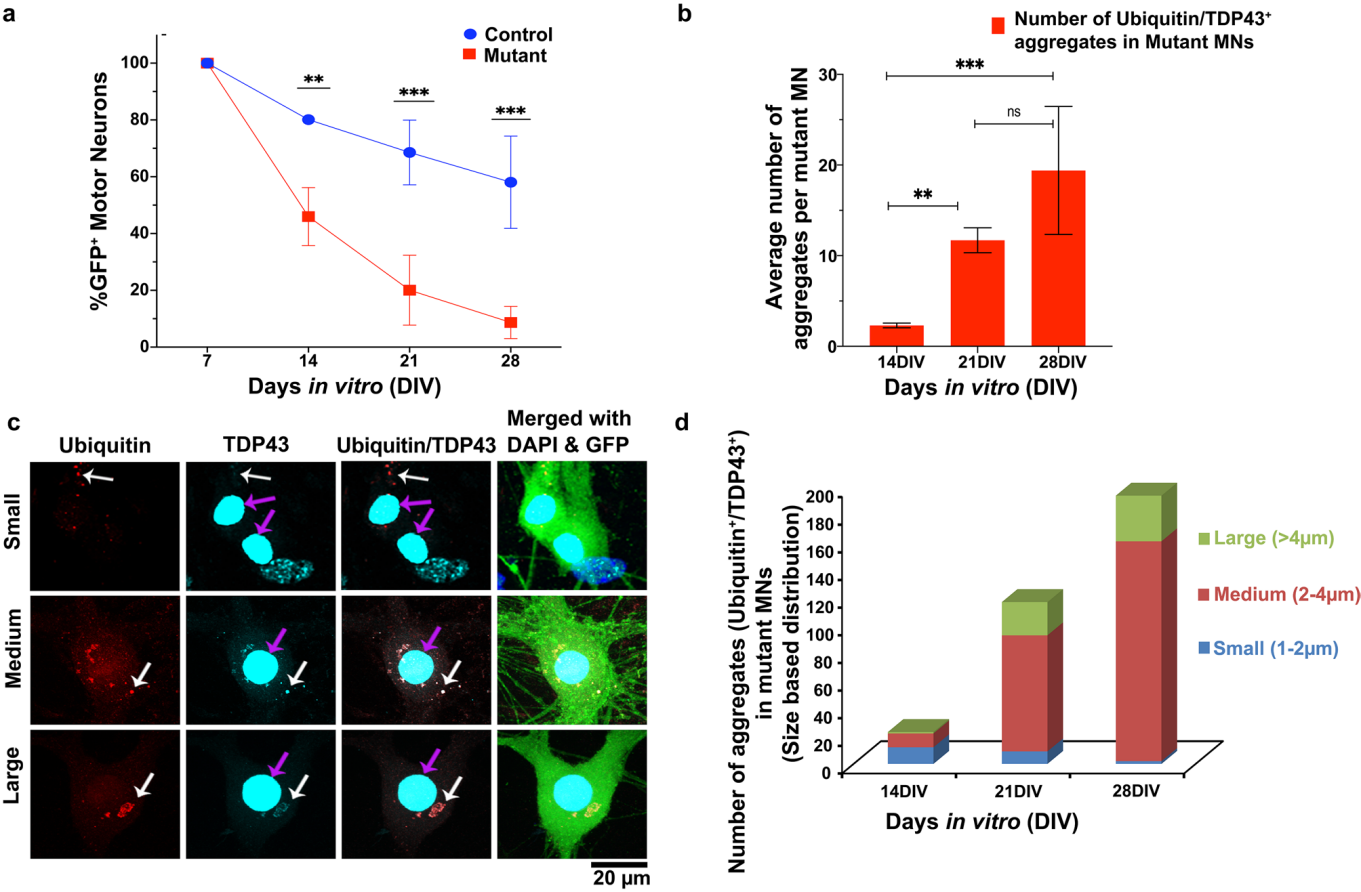
MN viability & quantification of cytosolic TDP-43 aggregates. (**a**) In vitro, quantification of MN viability showed a rapid decrease in the number of mutant MNs carrying TDP-43 mutation from 7 to 28DIV compared to that of control MNs without the mutation (p ≤ 0.001,n ≥ 3). All fluorescent intensities were normalized to 100% at 7 DIV. (**b**) Total number of Ubiquitin^+^/TDP-43^+^ aggregates per GFP^+^ mutant MNs increased from 14 to 28DIV (n = 10,p ≤ 0.001) (**c**) Immunocytochemical staining of TDP-43 & Ubiquitin and their classification into different sizes in the mutant MNs (GFP^+^). White arrows indicate the different sized cytosolic aggregates and pink arrows indicated the TDP-43 in the nucleus. (**d**) There is an increase in medium and large sized cytosolic TDP-43^+^/Ubiquitin^+^ aggregates in mutant MNs from 14 to 28DIV. P values: ^*Non significant (ns)*^*P* > 0.05, *P ≤ 0.05, **P ≤ 0.01, ***P ≤ *0.001.*

The overall MN viability was plotted as a function of culturing time days in vitro, for both mutant and control cells (Fig. 3a). As anticipated, the mutant cell line demonstrated a steep monotonic mortality through 28 DIV while the control cell line showed higher viability. The decrease in GFP^+^ control MNs by 28 DIV compared to 7 DIV (Fig. 3a) can be attributed to normal cell losses over time due to cell reprogramming, apoptosis, etc. occurring during normal cell development whereas the increased mutant mortality can be attributed to TDP-43 cytosolic toxicity imposed by the ALS phenotype. We also identified a linear increase (p ≤ 0.05, n = 10) in the average number of cytosolic ubiquitin^+^/TDP-43^+^ aggregates in the mutant line with time, correlating to mutant MN mortality (Fig. 3b). Further investigation showed a nominal size distribution with the cytosolic aggregates. The cytosolic TDP-43^+^/ubiquitin^+^ aggregates were categorized (Fig. 3c) into small (1–2 µm), medium (2–4 µm), and large (> 4 µm) aggregates. Figure 3d shows that there is an increase in the medium and large cytosolic TDP-43/ubiquitin aggregates at 21DIV and 28DIV in the mutant cell line presumably resulting from saturation and suggesting a potential interruption in the protein transport system.

**Figure 4:**
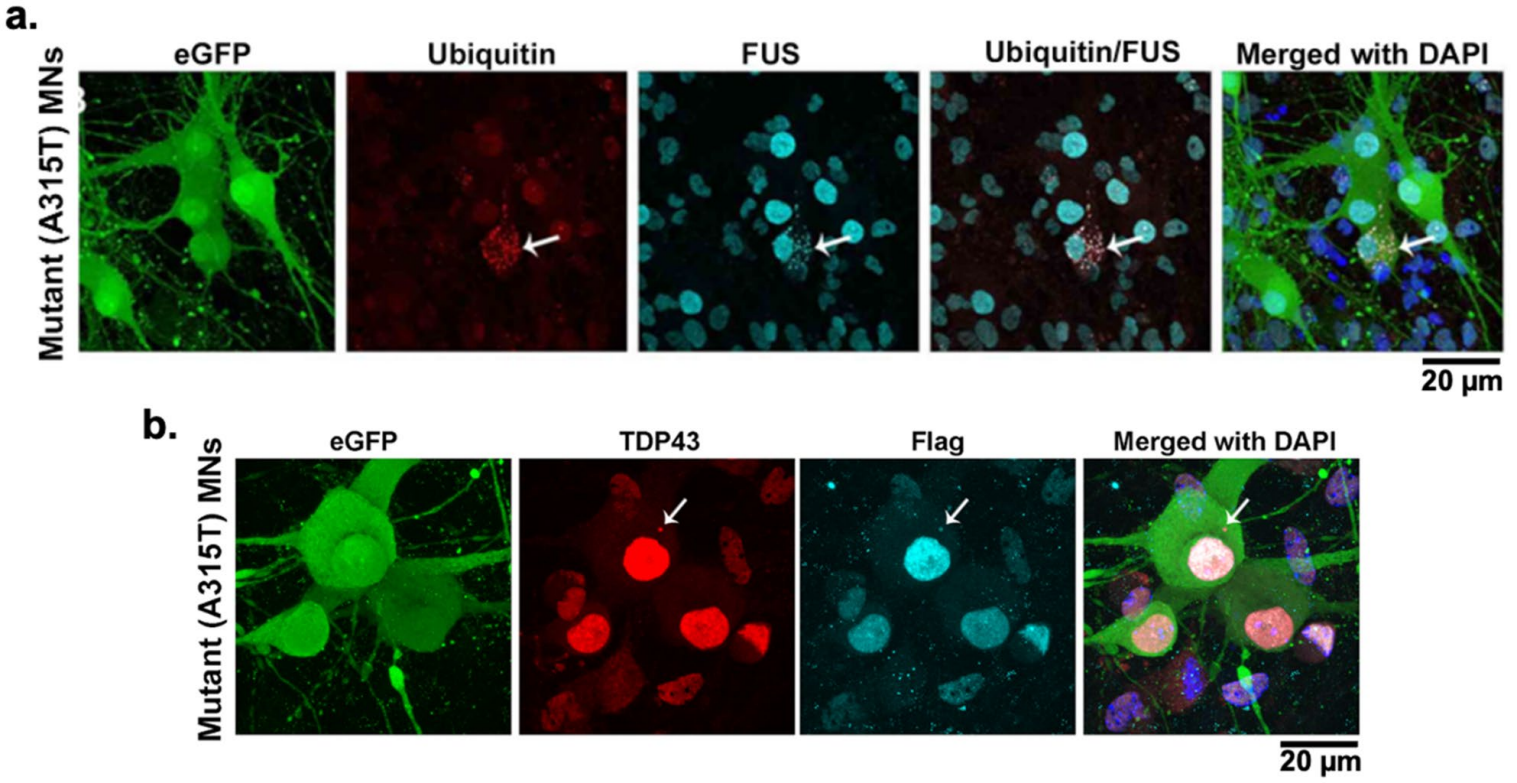
Validation of mutant TDP-43-A315T transgene as the source of cytosolic TDP-43 aggregates. (**a**) Immunocytochemical staining of mutant MNs. 32.2 ± 34.8% (n ≥ 5) of Mutant (A315T) cell-derived motor neurons also expressed FUS and co-localized with cytosolic ubiquitinated aggregates (indicated with arrows). (**b**) Immunocytochemical staining showing the cytosolic co-localized FLAG peptide with that of the TDP-43 (indicated with arrows), validating the generation and presence of TDP-43 in the mutant cells is due to the mutant gene.

FUS aggregates^23,24^ are another common protein mislocalization associated with the pathophysiology of familial ALS. However, the cause and action of FUS cytosolic mislocalizations are somewhat more ambiguous than TDP-43^23,49^. To determine whether mutant cell lines also show any FUS mislocalizations, we immunocytochemically stained mutant MNs for FUS and identified that 32.2 ± 34.8% (n ≥ 5) of cytoplasmic aggregates expressing TDP-43/Ubiquitin also expressed FUS (arrowed in Fig. 4a). Though this does not categorically identify the presence of a mutant gene or the cause of FUS mislocalization, it does indicate the presence of mislocalized cytoplasmic FUS protein along with TDP-43 and suggests a common aberrant biochemical pathway.

To validate that TDP-43 protein mislocalization resulted from the TARDBP mutation and not from an arbitrary ancillary effect, FLAG-Tag was used. FLAG-Tag is a polypeptide protein sequence added to the end of a targeted protein sequence, in this case at the end of the TDP-43 mutant gene sequence. FLAG is concurrently expressed with the associated TDP-43 gene and serves as a “tag” for TDP-43 expression (Fig. 4b). We found that in mutant MNs, FLAG also co-deposits with TDP-43/Ubiquitin cytoplasmic aggregates (arrowed in Fig. 4b), suggesting that the cytoplasmic TDP-43 aggregates were formed as a result of the expression of the TDP-43 tagged gene construct and not due to the native mouse TDP-43 expression. From western blot analysis (Figure S6a-d) we can confirm that the transgenic TDP-43 detected with the FLAG antibody was expressed only in mutants and not in control MNs (relative to β-tubulin) throughout the differentiation. Moreover, western blot analysis indicates that not only is there a decrease in total TDP-43 expression over time (both in control and mutant cells) but also the total TDP-43 expression in mutants is always lower than the control cells thereby ruling out the possibility of over-expression of the transgenic protein to be the cause of disease phenotype.

To rule out the possibility that the observed mutant phenotype was clone specific, we differentiated ES cells from a second independent mutant clone into motor neurons. As expected, we discovered that ES cell-derived motor neurons from the second clone with the TDP-43-A315T transgene also formed cytoplasmic aggregates that stained positive for both TDP-43 and ubiquitin, confirming that the cytoplasmic aggregates are due to the introduced transgene (Figure S2).

Taken together, this indicates that the mutant ALS phenotype cell line we generated strongly expresses the characteristic features of familial ALS, sharing ubiquitinated TDP-43 cytoplasmic aggregates, and is therefore an ideal candidate for studies of ALS pathophysiology.

### MN rescue using rapamycin

Protein aggregates are an important feature in ALS pathology and targeting them as a therapeutic treatment for ALS is a more promising^54^ approach than the current approaches that focus on delaying progression of the disease. One of the more promising approaches is the use of mTOR inhibitors such as rapamycin to clear cytosolic aggregates of TDP-43^32^. In the following sections, we investigated the efficacy of rapamycin to salvage ALS phenotypes.

#### Rapamycin treatment in a 2D culture

**Figure 5:**
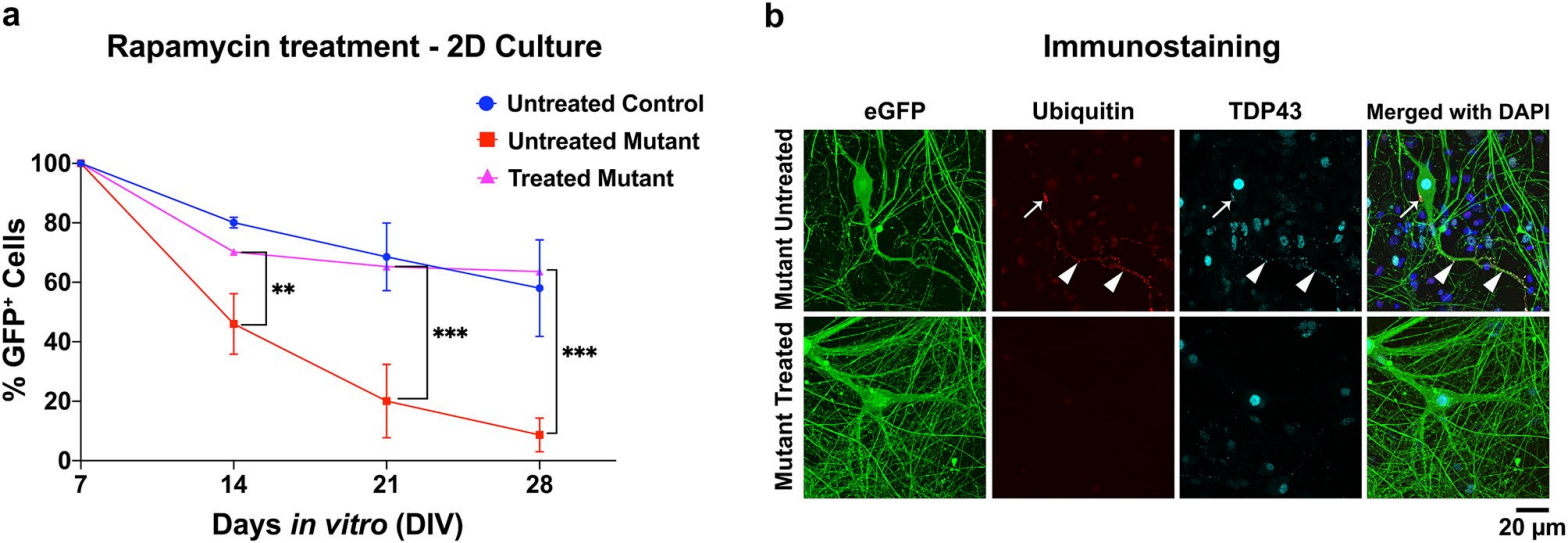
Rapamycin assay in 2D culture. (**a**) Mutant (A315T) MNs show an increase in survival when treated with 1 µM of rapamycin with %GFP^+^ cells equivalent to those of control at 21DIV and 28DIV. Mutant MNs not treated with rapamycin show a rapid decrease in survival from 7 to 28DIV compared to the control MNs(Untreated Control). All fluorescent intensities were normalized to 100% at 7 DIV. (**b**) Immunostaining of treated and untreated mutant MNs for cytosolic TDP-43 and Ubiquitin showing the reduction/absence of the cytosolic aggregates in the treated samples compared to that of untreated samples (arrows are indicating the cytosolic aggregates). P values: ^*Non significant (ns)*^*P* > 0.05, *P ≤ 0.05, **P ≤ 0.01, ***P ≤ *0.001.*

After fully differentiating both control and mutant ES cells into MNs for 7DIV using standard MN differentiation media, the cultures were then exposed to 1 µM rapamycin and MN viability was monitored at 14DIV, 21DIV and 28DIV. Mutant cells (ALS phenotype), treated with rapamycin showed significantly improved MN survivability compared to the untreated mutant cells (e.g., comparing treated mutants vs. untreated mutants by 28 DIV, a 54.91 ± 5.67% increased MN survivability was observed with a *p* ≤ *0.001 & n* ≥ *3* by two-way ANOVA-sidak test). In fact, rapamycin treated mutant MNs demonstrated essentially equivalent viability as untreated control MNs (e.g., by 28 DIV the difference in MN viability was only 5.54 ± 16.9% with a *p* > *0.05 & n* ≥ *3* by two-way ANOVA-sidak test) indicating that 1 µM rapamycin treatment is capable of fully rescuing ALS phenotypes (Fig. 5a, for detailed statistical analysis and comparison see supplementary Sheet 1). Further examination of 1 µM rapamycin treated cells revealed the mitigation of cytosolic ubiquitinated TDP-43 aggregates (Fig. 5b). This is in direct contrast to the presence of the cytosolic aggregates in the untreated MNs (indicated by arrows).

#### Rapamycin treatment in a 3D culture

The above experiments effectively demonstrate the potential of rapamycin to rescue the ALS MN phenotypes. However, recent reports^55^ suggest that traditional 2D cell plating in a homogenous media may fail to fully reproduce a true in vivo environment. In contrast, 3D cell cultures generally preserve inherent morphological and phenotypical cell development and growth more faithfully. To ameliorate deficiencies imposed by 2D cell cultures, a number of successful 3D culturing techniques have surfaced, most notably embryoid bodies, gel matrices and scaffolding. Here we adopt a microfluidic approach to generate rapamycin concentration gradients within a 3D gel matrix and evaluate the permissive growth zones for the ALS phenotype as a function of the spatial rapamycin concentration. This microfluidic approach enables a rich experimental platform that not only facilitates faster, more robust and faithful developmental and drug testing, but more importantly, enables uniquely new experimentational venues for exploration. Yet, a significant drawback to the microfluidic approach is an inability to obtain high resolution micrographs of subcellular geometries due to inherent light scattering and absorptive effects in thick, multilayers cellular samples that are firmly ensconced in an extracellular matrix. For this reason, we employed traditional 2D culturing to clearly visualize subcellular features, while capitalizing on the facile, quick and contextually relevant 3D environment of microfluidics to provide a more detailed and comprehensive investigation into ALS: its cause and its remediation.

##### Characterization of microdevice

A full explanation outlining the generation of 3D concentration gradients is presented in Demers.et.al^43,44^. In brief, a traditional source/sink diffusion motif is employed to maintain a steady-state concentration profile across a cell culture. Rapamycin is introduced to one side of the microdevice cell chamber at a high constant concentration (source) and allowed to freely diffuse to a lower concentration (sink) on the opposite side of the microdevice cell chamber (Figure S3a). For 1D diffusion at steady state, this results in a linear rapamycin concentration across the microdevice cell chamber from source to sink. This spatial gradient is then used to assess ALS cells viability at different positions (bin) within the gradient with each bin corresponding to a specific rapamycin concentration.

Computational modeling of the rapamycin concentration profile was performed using COMSOL Multiphysics, a finite element analysis program (Figure S3a) to assess and quantify gradient performance. Experimental confirmation of the in silico results were obtained by establishing a linear fluorescein concentration gradient across the gel filled chamber of the microdevice and quantifying the fluorescent profile by measuring the fluorescent intensity at 520 nm (excitation 488 nm) as a function of distance across the chamber (Figure S3b). At short diffusion times, profiles followed normal Cottrell distributions that transitioned into a linear, steady-state gradient in about 10–20 min. The linear profiles were stable indefinitely, as long as the fluorescein concentrations at the two boundaries (source and sink) were continually refreshed with reagent flow from the microchannels, i.e., boundary concentrations kept constant (Figure S3b).

##### Rapamycin assay in the microdevice

**Figure 6:**
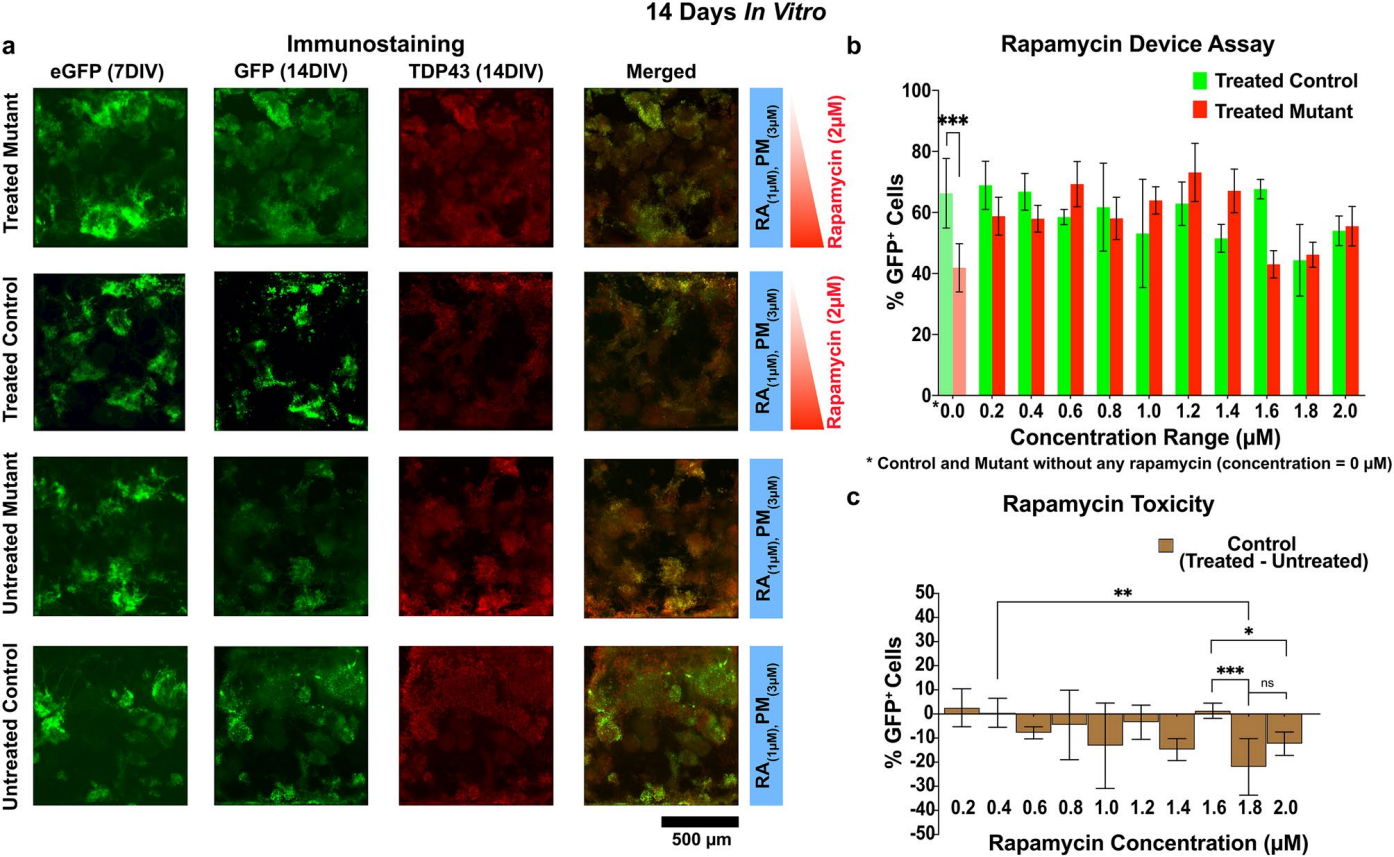
Rapamycin assay in microdevice (14DIV). (**a**) Typical microdevice immunostaining for GFP and TDP-43 at 14DIV under a rapamycin gradient. Red sidebars immediately to the right indicate the spatial direction of the rapamycin gradient (red: 0 –2 µM) while the blue bars indicate a constant concentration of culture media morphogens. (**b**) Plots the average (n ≥ 4) percent viability of rapamycin treated MNs (GFP^+^) i.e. viability of treated mutant MNs (red) and treated control MNs (green) as a function of rapamycin concentration. *Indicates both mutant and control MNs which are not exposed to rapamycin, i.e. untreated MNs respectively. (**c**) Shows the relative MN toxicity of rapamycin on control MNs, i.e. the percent viability of rapamycin treated control MNs minus the average viability of rapamycin untreated control MNs. A positive ( +) value indicates an increased survival of control MN’s without rapamycin and a negative (−) value indicates a toxicity effect of rapamycin. P values: ^*Non significant (ns)*^*P* > 0.05, *P ≤ 0.05, **P ≤ 0.01, ***P ≤ *0.001.*

**Figure 7:**
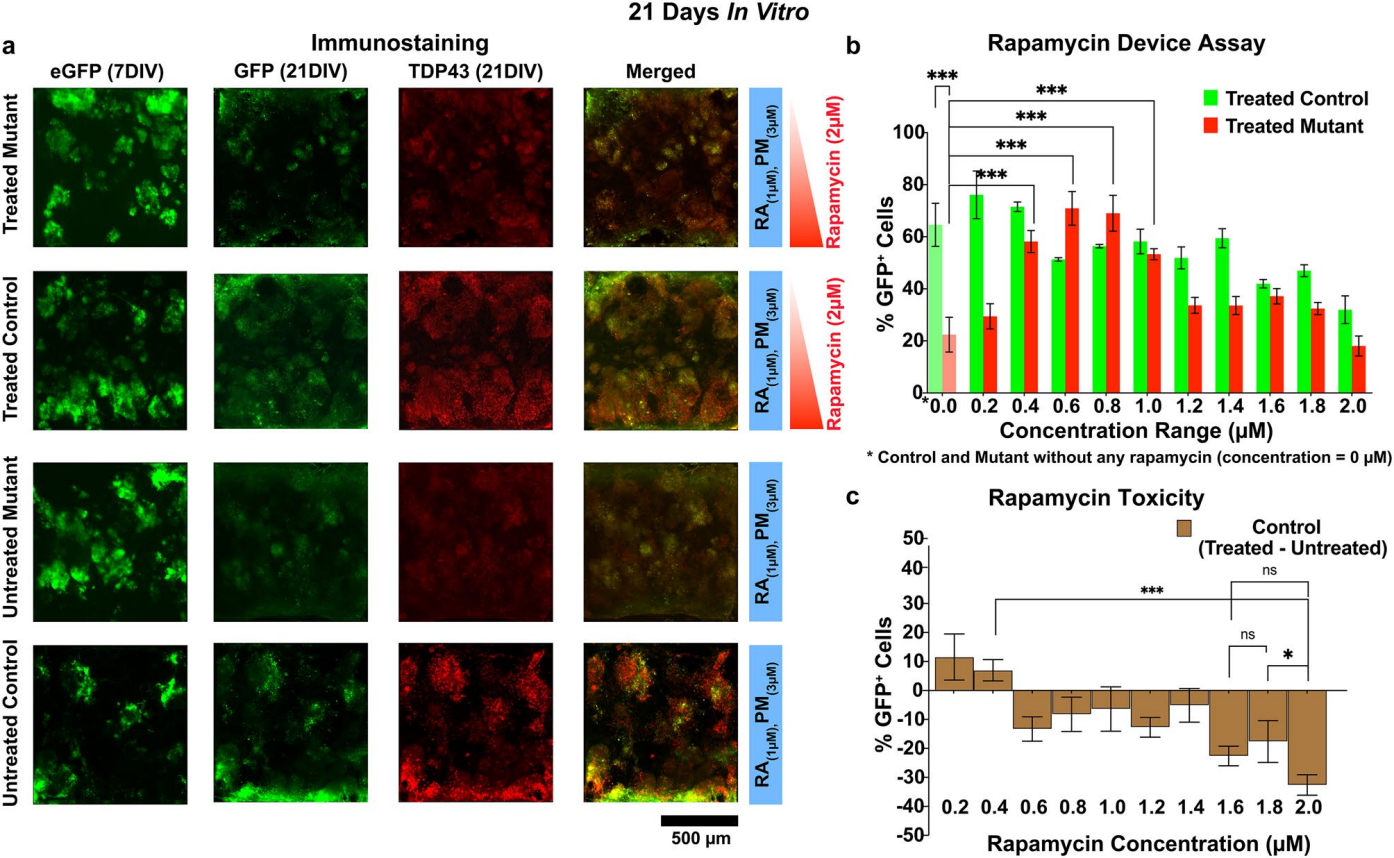
Rapamycin assay in microdevice (21DIV). (**a**) Typical microdevice immunostaining for GFP and TDP-43 at 21 DIV under a rapamycin gradient. Red sidebars immediately to the right indicate the spatial direction of the rapamycin gradient (red: 0–2 µM), while the blue bars indicate a constant concentration of culture media morphogens. (**b**) Plots the average (n ≥ 4) percent viability of rapamycin treated MNs (GFP^+^) i.e. viability of treated mutant MNs (red) and treated control MNs (green) as a function of rapamycin concentration. *Indicates both mutant and control MNs which are not exposed to rapamycin, i.e. untreated MNs respectively. (**c**) Shows the relative MN toxicity of rapamycin on control MNs, i.e. The percent viability of rapamycin treated control MNs minus the average viability of rapamycin untreated control MNs. A positive ( +) value indicates an increased survival of control MN’s without rapamycin and a negative (−) value indicates a toxicity effect of rapamycin. P values: ^*Non significant (ns)*^*P* > 0.05, *P ≤ 0.05, **P ≤ 0.01, ***P ≤ *0.001.*

ES cells, both control and mutant, were differentiated into MNs for 7 DIV in the microdevice using standard MN differentiation media according to protocols outlined in the methods Sect. (4.4). A MN cell identity was confirmed by expression of the Hb9::GFP reporter (eGFP). At 7DIV, the MNs were then exposed to a linear rapamycin gradient for the remaining duration of the experiment by introducing 2 µM rapamycin into the source media and 0 µM rapamycin in the sink media. Immunostaining of MNs was carried out using anti-GFP and anti-TDP-43 antibodies at 14DIV and 21DIV (Figs. 6a & 7a). GFP was used as a marker for differentiated motor neurons and anti-TDP-43 was used to stain TDP-43 aggregates. GFP^+^ fluorescent intensities were plotted as a function of rapamycin concentration, i.e., the spatial position in the microdevice. Intensities were averaged over five samples and normalized to eGFP fluorescent intensities at 7DIV, i.e. all average eGFP intensities at 7DIV were normalized to 100%.

By 14 DIV the untreated mutant MNs demonstrated a 24.4 ± 13.8% (*p* ≤ *0.001, n* ≥ *4*, two-way ANOVA-sidak test) lower viability than their untreated control MNs as indicated in Fig. 6b, 0 µM rapamycin. Again, a general loss of untreated MN viability with time is expected and attributed to normal cell losses during early MN development and growth. It is encouraging that at 14DIV rapamycin treated mutant cells showed virtually complete rescue over all rapamycin concentrations when compared to the corresponding treated control MNs (Fig. 6b). This is also consistent with the absence of TDP-43 cytoplasmic aggregates in rapamycin treated 2D MN cultures (Fig. 5b). However, a general MN toxicity begins to emerge which is shown clearly in Fig. 6c. Here the relative percentage of viable control MNs as a function of rapamycin concentration is plotted for 14 DIV using the concentration of untreated control (0 µM rapamycin) as a reference, i.e. percent viability for rapamycin treated control MNs minus untreated control MNs. Generally, little to no rapamycin sensitivity is seen at low rapamycin concentration for shorter exposure times (≤ 14DIV), but for rapamycin concentrations > 1 µM, a small but noticeable decrease in MN viability begins to emerge (e.g., comparing MN viability at 0.4 µM to 1.8 µM in Fig. 6c, a 21.47 ± 14.3% decrease in MN viability is observed, with a *p* ≤ *0.01 & n* ≥ *4* by a two-way ANOVA-sidak test) and becomes more pronounced at longer rapamycin exposure times, see 21 DIV (Fig. 7c).

As cells progressed to 21DIV, it was not surprising to see a 42 ± 10.652% (*p* ≤ *0.001,n* ≥ *4*, two-way ANOVA-sidak test) increase in mortality for untreated mutant MNs compared to the untreated control MNs (Fig. 7b, 0 µM rapamycin). However, several interesting trends also surfaced. First, low rapamycin concentrations (≤ 0.2 µM) were ineffectual in salvaging mutant MNs, most likely due to reduced inhibition of the mTOR pathways and/or suppressed TDP-43 clearing, resulting in significant TDP-43 cytoplasmic depositions. This is not surprising as most therapeutic drugs have threshold concentration limits, both high and low. Second, rapamycin concentrations between 0.4 to 1.0 µM presented a permissive MN growth zone for both control and mutant MNs. Over this region rapamycin treated mutant MNs showed a dramatical increase in viability, 40.44 ± 11.12% (*p* ≤ *0.001, n* ≥ *4,* two-way ANOVA-sidak test) in comparison to untreated mutant MNs (Fig. 7b, 0 µM rapamycin), while control cells demonstrated little to no adverse effects from rapamycin exposure. Third, at high rapamycin concentrations > 1.4 µM, control MNs experienced a substantial decrease in MN viability (e.g., comparing MN viability at 0.4 µM to 2.0 µM in Fig. 7c, a 26.1 ± 13.9% decrease in MN viability is observed, with a *p* ≤ *0.001 & n* ≥ *4* by a two-way ANOVA-sidak test), indicating increased toxicity from 14 to 21DIV (Fig. 7c, compared with Fig. 6c, Sheet S1 for two-way ANOVA group comparisons), and while rapamycin treatment still salvaged mutant MNs from demonstrating the ALS phenotype, mutant MNs also showed a steady loss of viability at higher rapamycin concentrations (Fig. 7b, compare with Fig. 6b).

Nevertheless, comparing the TDP-43 aggregates per MN at different time interval (Fig. 3b) with an observed increase in untreated MN mortality from 2D (Fig. 5a) and 3D (Figs. 6a, 7a) MN cultures indicate a possibility that the cytosolic TDP-43 aggregates are related to the increased mortality in mutant MNs and rapamycin was able to rescue these mutant MNs from degeneration in both 2D and 3D cultures indicating a potential path for ALS remediation.

## Conclusion

In this article mouse embryonic stem cells possessing the mutant human ALS TDP-43-A315T transgene were successfully derived and differentiated into MNs under both 2D and 3D environments to assess the efficacy of rapamycin as a rescue to the ALS phenotype. A traditional 2D cell culture does not fully represent a true in vivo environment, and a 2D assay is usually laborious and less economical. A 3D gradient microfluidic^56–58^ assay overcomes these limitations but has its drawbacks such as, difficulty in achieving high resolution imaging to differentiate intracellular components due to the dense 3D cell clusters. Therefore, by merging a traditional 2D cell culturing approach with a 3D microfluidic platform, we were able to combine the advantages inherent in both techniques (high-resolution 2D images with high-throughput 3D spatial titrations) to more comprehensively, accurately and quickly assay MN development and degeneration under appropriate pharmacological remediation (rapamycin). Using this combined approach, we demonstrated motor neurons from the mutant cell line showed cytosolic mislocalization of TDP-43 aggregates and FUS protein with ubiquitin co-deposition, representing the hallmark phenotypical ALS pathology. Moreover, it was confirmed that mislocalization of TDP-43 aggregates due to the mutant phenotype is not clone specific and occurs in other variants possessing the same ALS mutation. Additionally, we were able to show that TDP-43 mislocalization increases with culturing time and is correlated to cell mortality at later life cycles.

Using the microfluidic platform, we also demonstrated an optimal 40.44% rescue of the ALS phenotype over a concentration range of 0.4–1.0 µM. For lower rapamycin concentrations, the drug was ineffectual in rescuing the ALS phenotype while rapamycin demonstrated significant toxicity for higher concentrations and exposure times. This strongly suggests potential remediation of ALS is possible using mTOR inhibitors such as rapamycin. An essential advantage to the microfluidic assay presented here is that an entire rapamycin titration was performed in a single cell culture experiment (with replicates), thereby greatly enhancing test throughputs and results. The small size of the device, 1 mm × 1 mm, allows for parallel testing of large device arrays increasing the testing throughput, thereby enabling drug discovery and screening, as well as patient-specific drug screening and treatment. Due to the intrinsic potential of the microfluidic device to differentiate cells in 3D and retain their spatial and morphological construct similar to in vivo, in future, a modified design of this current platform can be utilized in exploring and understanding axonal progression and degeneration over time in ALS cell lines (mESCs and human IPSCs) while performing a therapeutic screening/treatment.

## Materials and methods

### Derivation of transgenic (mutant) mouse embryonic stem cells

Embryonic stem (ES) cells possessing both ALS phenotype mutations in the TARDBP gene and a Hb9::GFP reporter were derived from crossing B6.Cg-Tg (Prnp-TARDBP*A315T) 95Balo/J mice with B6.Cg-Tg (Hlxb9-GFP)1Tmj/J mice (The Jackson Laboratory, Bar Harbor, ME, USA (JAX)). Enhanced GFP (green fluorescence protein) is expressed under the control of the mouse Hb9 promoter, and thus, eGFP expression can be used to identify putative motor neurons^59^. Following derivation, B6.Cg-Tg(Prnp-TARDBP*A315T) 95Balo/J:B6.Cg-Tg(Hlxb9-GFP) 1Tmj/J ES cells (referred to as mutant ES cells or mutant (A315T)) were cultured using standard ES cell media with 2 inhibitor supplements (2i)^60^. The ES cells were genotyped and following karyogram analysis, they were characterized with standard pluripotent markers (Figure S1a-b). Motor neurons differentiated from these cells are referred to as mutant MNs. Human mutant transgene is referred as TDP-43-A315T.

The ES cells obtained from B6.Cg-Tg (Hlxb9-GFP)1Tmj/J mice having only the Hb9::GFP reporter are referred to as control ES cells and differentiated MNs from these ES cells are referred to as control motor neurons. All these cell lines are isogenic as they were derived from littermates that are inbred strains of mice. The mice are > 99.99 percent identical as they are maintained on an inbred C57BL/6 J genetic background by a continuous brother, sister mating at The Jackson Laboratory. The mice used in the generation of the cell lines were treated in accordance with Animal Research: Reporting of In Vivo Experiment (ARRIVE) guidelines.

All mouse husbandry and procedures were reviewed and approved by the Institutional Animal Care and Use Committee at The Jackson Laboratory and were carried out according to the NIH Guide for Care and Use of Laboratory Animals (AUS# 01006).

### Mutant and control motor neuron development

All embryonic stem cells were differentiated in MN differentiation media^61^ and cultured at 37 °C under 5% CO_2_. The MN differentiation media contained: Advanced DMEM/F12 and Neurobasal media in a 1:1 ratio supplemented with B2, N27, 5% heat-inactivate horse serum (Invitrogen), 10 ng/ml of CNTF, GDNF, BDNF, and NT-3 (R&D Systems), 3 µM sonic hedgehog agonist purmorphamine (Calbiochem, MilliporeSigma) and 1 µM retinoic acid (RA, Sigma).

The experimental protocol for the development of ALS MNs is generally divided into two independent procedures: (a) a traditional 2D culturing of motor neurons and (b) a 3D culturing of MNs in a fluidic microdevice. Using the appropriate 2D or 3D protocols, all ES cells were cultured for 7 days in vitro (DIV) in MN differentiation media before exposing them to rapamycin. This time frame was sufficient to differentiate ES cells into MNs, as evidenced by Hb9::GFP expression.

### 2D culturing of ES cells into MNs

Embryonic stem cells were first grown to embryoid bodies for 3 days in MN differentiation media using standard embryoid body culturing techniques^62,63^. Embryoid bodies were resuspended in fresh differentiation media and allowed to culture for an additional 2 days. Embryoid bodies were then collected and dissociated into single cells using papain (Worthington Kit). Dissociated cells were then plated on coverslips coated with reduced growth-factor Matrigel (BD Biosciences, Bedford, MA) and the individually coated coverslips were placed in a 24-well microtiter plate containing MN differentiation media and allowed to grow. Dissociated cells were plated at a density of 20,000 cells/well. Motor neuron culture media was changed twice a week.

### 3D culturing of ES cells in the microfluidic device

Details of the microdevice construct and experimental conditions are published in a previous report^43^. Experimental parameters deemed necessary for completeness and/or analyzing the results are repeated here. Embryonic stem cells (Control or Mutant) were suspended in Geltrex (Gibco, LDEV-Free, hESC-Qualified, Reduced Growth Factor Basement Membrane Matrix) at 4 °C at a density of 10^6^–10^7^ cells/ml. 0.3 µl of cell-ladened gel matrix was dispensed into the microfluidic device culture chamber and allowed to set by warming to 37 °C. Because of the small sample volume, both gel matrix cooling and heating could be performed within several seconds, minimizing stress to the ES cells.

Fluidic connections to the microdevice were immediately established to continuously perfuse the embryonic stem cells with the appropriate media (MN differentiation media and/or rapamycin). All media was pre-equilibrated with air at 5% CO_2_ and 37 °C.

### Chemical gradient generation

Chemical gradient generation capability of the microdevice was validated using fluorescein according to previously published procedure^43^. In brief, the microdevice cell chamber was loaded with Geltrex. Fluorescein (5 µM) was introduced to one fluidic channel of the microdevice, while DI water was introduced into the opposite microchannel. A concentration gradient was thereby established between the microchannels by diffusion of fluorescein (diffusion coefficient: 4.9 × 10^−6^ cm^2^ s^−1^) from source to sink. The concentration profile of fluorescein generated in the cell chamber was captured using Zeiss Axio Observer Z1 inverted microscope and fluorescent emission intensities at 520 nm (excitation wavelength 488 nm) were mapped as a function of distance to verify the creation of a linear source/ sink concentration profile within the gel matrix (see Figure S3).

### Therapeutic drug interventions (Rapamycin)

By 7DIV, ES cells had developed into MNs as evidenced by expression of Hb9::GFP. At 7DIV, both 2-D and 3-D perfusion media were changed to include rapamycin. For 2-D cultures, rapamycin (1 µM) was added directly to MN differentiation media and allowed to incubate in the 24 well microtiter plate. For 3-D microfluidic cultures, a rapamycin diffusion gradient was established across the cell culture chamber by addressing one microfluidic channel with MN differentiation media containing 2 µM rapamycin while addressing the second microchannel with only MN differentiation media (0 µM rapamycin). Flow rate in each channel was kept constant at ~ 100 µl/hr for the duration of the experiments. This maintained a linear rapamycin gradient across the MN culture with 2 µM rapamycin at the high end and 0 µM rapamycin at the low end, thereby exposing MN cultures to a range of rapamycin concentrations in a single device. The precise concentration that each MN experiences is a function of its spatial position within the microdevice chamber.

### Immunocytochemistry & western blotting

Following 7, 14, 21, 28 DIV, ES cell-derived motor neuron cultures were fixed using 4% paraformaldehyde in PBS for 10 min followed by several washes in PBS and incubated with antibodies against TDP-43 (1:500; Proteintech # 66734-1-Ig), TDP-43 (1:500, Proteintech # 1078-2-AP), ubiquitin (FK2 clone; 1:1000; MilliporeSigma # ST1200), GFP (1:500; Aves Labs, # 75-131), GFP (1:500; Sigma # A1112), Flag (1:500; Sigma # F3165), and FUS (1:500; Novus Biologicals # NB100-565) in PBS containing 10% goat serum at 4° C overnight. The cells were subsequently washed with PBS, incubated with Alex Fluor 488, 594, 637 secondary antibodies (1:500; Invitrogen) at room temperature (RT) for 2 h, washed, and then mounted in VECTASHIELD antifade mounting medium (Vector Laboratories). 2D images were captured using a Leica confocal microscope (Leica). 3D images were captured using Axio Observer Z1 (Carl Zeiss, Inc.) inverted microscope at 5 × magnification. Using standard western blot protocols, lysates were prepared from MNs (control and mutant) collected on 7, 14, 21, and 28 DIV and assayed for Flag (Sigma # F3165) and TDP-43 (Proteintech # 66734-1-Ig) proteins with β-tubulin as loading control. Blots were stripped and re-probed for each antibody against loading control.

### Image analysis and quantification in microdevices

For image analysis and quantification, the chamber was divided vertically into ten 100 µm zones (permissive zones or bins) along the gradient. Each zone represents an average range of rapamycin concentration based on a linear concentration profile. Fluorescence intensity, corresponding to the number of positive cells (in our case, motor neurons) was plotted against the rapamycin concentration. GFP^+^ cells were counted using ImageJ (NIH). As individual cells were not resolved at the low magnification (5x), fluorescent intensities were used to quantify cells. An ‘unsharp mask’ was applied, followed by cell count (particle analysis). A native eGFP response on 7DIV(day-7) was used as the baseline for GFP expression. ANOVA in conjunction with an appropriate multiple comparison test was performed for statistical significance (for a detailed comparison, see Sheet S1 ). A high resolution imaging for the localization of nuclear TDP-43 to that of cytoplasmic TDP-43 without destroying the positional construct (gradient zones) was not possible in the microdevice samples (Figs. 6, 7a). The reason for this is, high resolution images are rather difficult to obtain in thick 3D cultures, even using confocal or epifluorescence techniques due to constraints imposed by light scattering and absorptive processes. This, as well as the propensity of ESCs to cluster, is one of the disadvantages of the 3D microfluidic approach and why high-resolution images were only provided for the planar 2D cultures. A higher magnification image of a typical MN cluster in the microdevice showing their morphology is indicated in supplementary figure S4. This scattering/clustering does not preclude quantitative assessments of the total fluorescence or expression, which is the metric used in the 3D microdevice analysis as outlined and referenced in our previous publication^43^.

## Supplementary Information

Supplementary Information 1.

Supplementary Information 2.
