# Author Correction: A microfluidic approach to rescue ALS motor neuron degeneration using rapamycin

**DOI:** 10.1038/s41598-021-99383-w

**Published:** 2021-09-29

**Authors:** Phaneendra Chennampally, Ambreen Sayed-Zahid, Prabakaran Soundararajan, Jocelyn Sharp, Gregory A. Cox, Scott D. Collins, Rosemary L. Smith

**Affiliations:** 1 Microinstruments and Systems Laboratory, University of Maine, Orono, ME 04469 USA; 2 Graduate School of Biomedical Sciences and Engineering, University of Maine, Orono, ME 04469 USA; 3 The Jackson Laboratory, Bar Harbor, ME 04609 USA; 4 Department of Chemistry, University of Maine, Orono, ME 04469 USA; 5 Department of Electrical and Computer Engineering, University of Maine, Orono, ME 04469 USA

Correction to: *Scientific Reports* https://doi.org/10.1038/s41598-021-97405-1, published online 13 September 2021

In the original version of this Article Phaneendra Chennampally and Ambreen Sayed-Zahid were omitted as equally contributing authors.

The original Article has been corrected.

