# Supplementary material for: A microfluidic approach to rescue ALS motor neuron degeneration using rapamycin
Source: Sci Rep. 2021 Sep 13;11:18168. doi: 10.1038/s41598-021-97405-1 (PMC8438029; doi:10.1038/s41598-021-97405-1)
Supplement: Supplementary file 2 — Supplementary Information 2. [file 41598_2021_97405_MOESM2_ESM.docx]

Supplementary Figure 1

Figure S1: mESC culture and MN formation in 2D. a) Standard ESC cell cultured in ESC media (cultured colonies) b) Genotyped, and following karyogam analysis, were characterized with standard pluripotent marker c-d) Embryoid formation and differentiation to motor neuron.

**
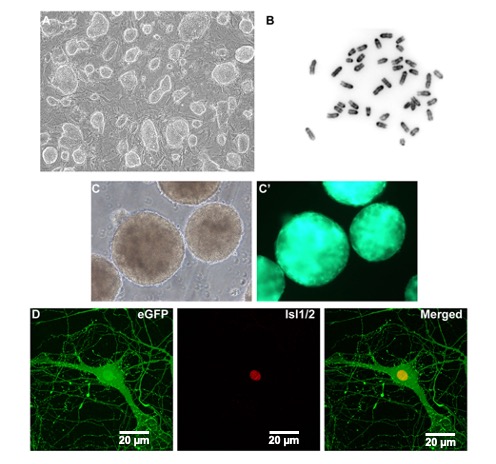
**

Supplementary Figure 2:


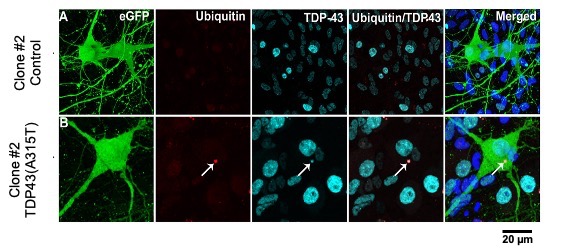


Figure S2: Immunocytochemical analysis of Clone #2. Clone #2 (second independent clone) mutant MNs having the TDP43-A315T mutant gene show the presence of cytoplasmic TDP43 and cytosolic ubiquitin aggregates.

Supplementary Figure 3


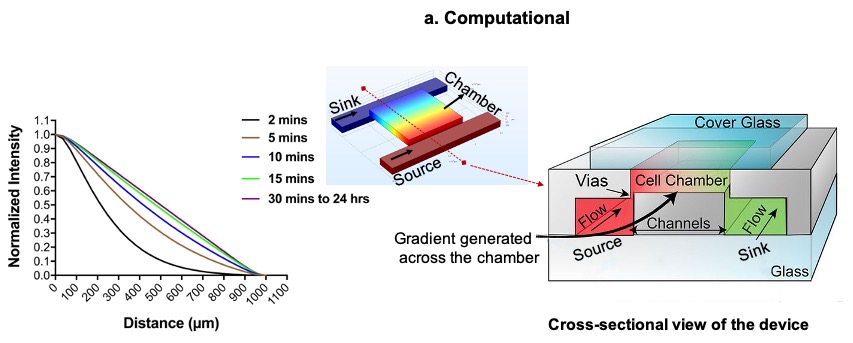


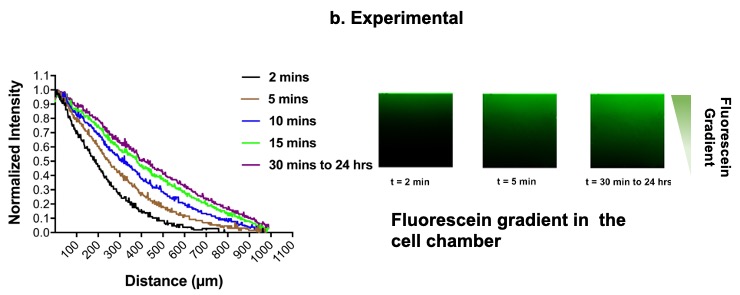


Figure S3: Validation of diffusion profiles in the microdevice. a) COMSOL simulations of a developing fluorescein diffusion profile as a function of time. Initial diffusion profiles follow the general Cottrell equation, but quickly transition into a linear, steady-state profile with time. Intensities are normalized to 1.0 for a source fluorescein concentration and 0 for the sink fluorescein concentration. Inset, middle, shows the source/sink configuration with the calculated diffusion profiles using a diffusion coefficient of 4.9 × 10^−6^ cm^2^ s^−1^ for fluorescein. A cross-sectional view of the microdevice illustrating the various features of the device (reproduced with permission from Demers^40^ et.al). Cell culture media flows through the “channels” and diffuses through the “Vias” into the “Cell chamber”. b). Experimentally measured diffusion profiles using the optical emission of fluorescein at 520nm. Micrographs to the right show a developing diffusion profile across the microdevice chamber (1mm x 1mm) at various times. A uniform linear diffusion gradient is clearly evident post 30min of diffusion and remains linear for as long as the source and sink concentrations are maintained in this case 24hrs before the experiment was terminated.

Supplementary Figure 4:


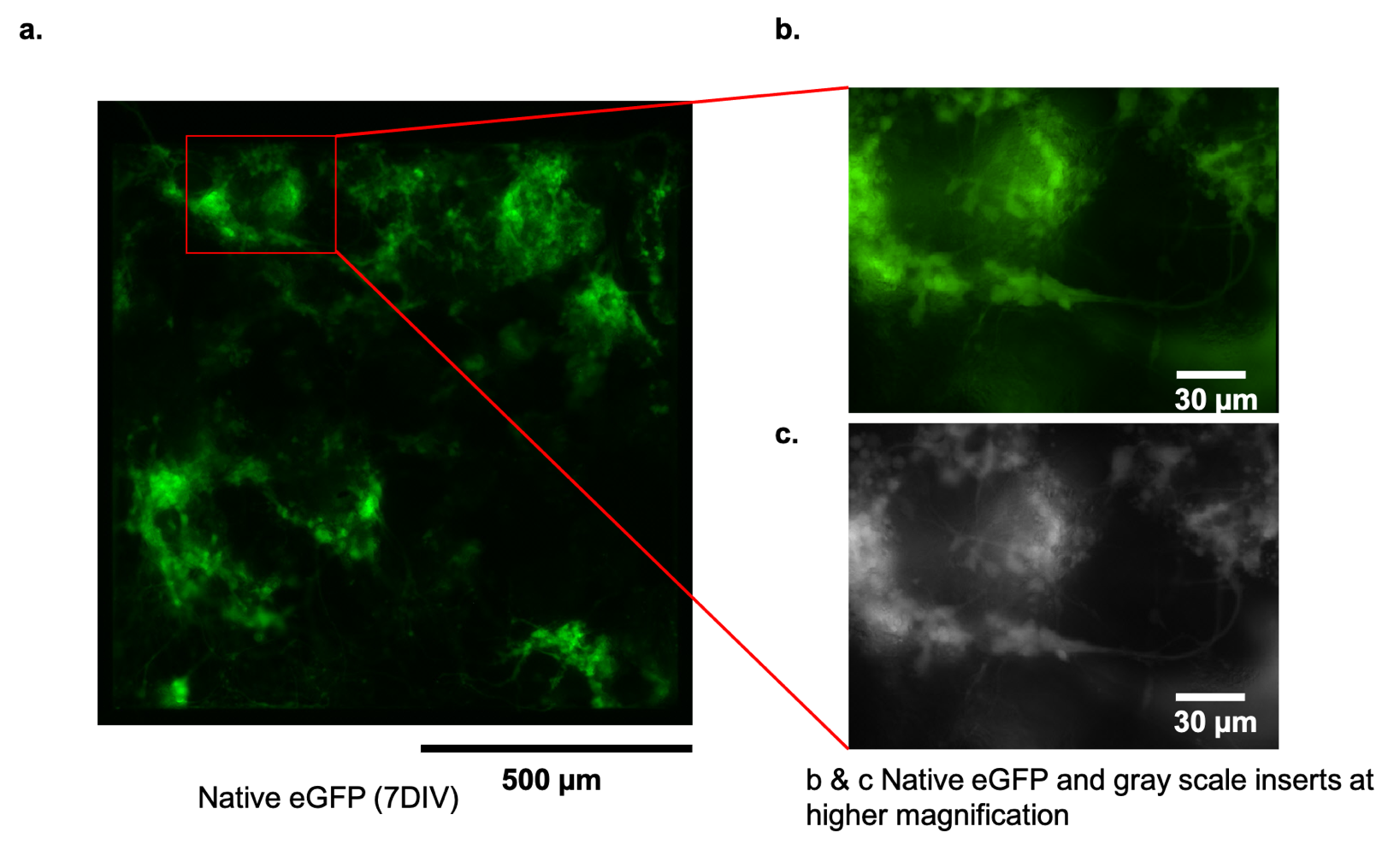


Figure S4: a) Low magnification image of native eGFP expressing differentiated motor neurons at 7 days *in vitro* in the microdevice. ESC’s are known to differentiate in MN clusters containing many cells and intertwined neurites that extend over a large depth of focus making meaningful high-resolution imaging of single cells and/or subcellular features difficult. b & c) Green colored (eGFP) and gray scale images of MNs respectively at higher magnification. MN somas and neurites are visible, but not clearly imaged due to light scattering and blurring from multiple focal planes. Generally, 2D are used to obtain high resolution cellular/subcellular images, while 3D imaging is used to obtain spatial cell distributions and fluorescent intensity averages.

Supplementary Figure 5:


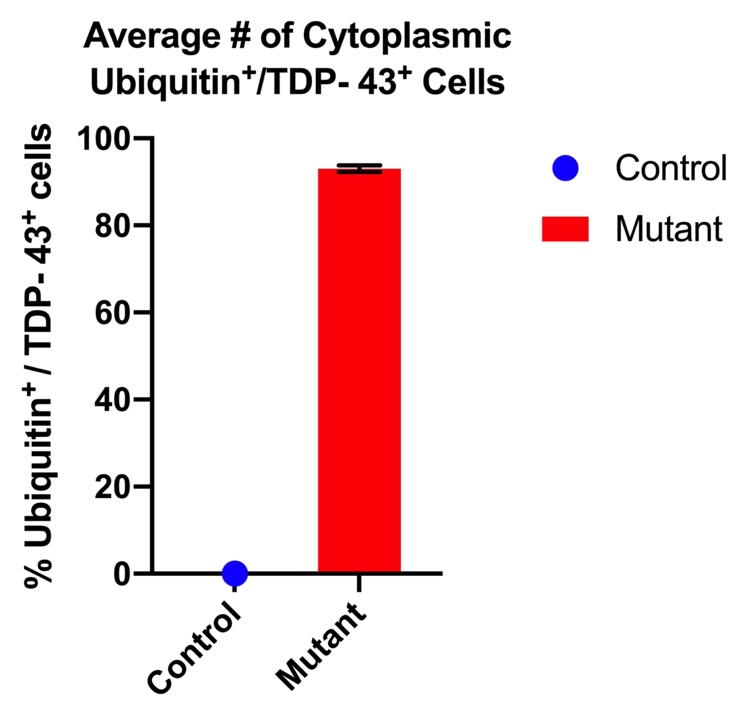


Figure S5: Average number of cells positive for cytoplasmic aggregates in control and mutant cells. Four plates (N=4) for each cell line (control and mutant) were analyzed for TDP-43/ubiquitin positives cells. In case of mutant cell line an average of 93.05±0.722 % (n_total_=97 cells) were positive for cytoplasmic TDP43/ubiquitin aggregates, whereas the average number of control (n_total_=169 cells) MNs positive for cytoplasmic TDP43/ubiquitin remained zero (0 %) between 7 DIV to 28DIV.

*At 7DIV or (1DIV to 7 DIV) the percent of control (n_total_=77 cells) and mutant (n_total_=43 cells) cells positive for cytoplasmic aggregate was zero. This data is considered as baseline and not included in the above graph.

Supplementary Figure 6:


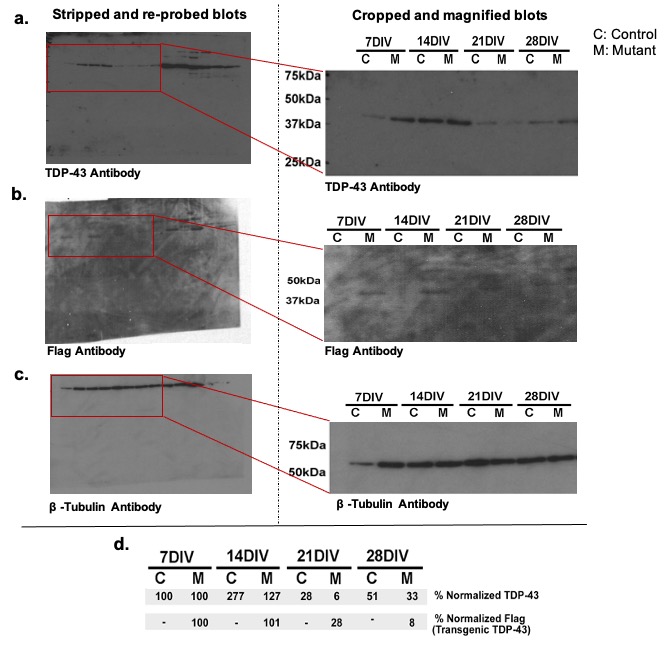


Figure S6: Western blot analysis was performed using lysates of both control and mutant motor neurons at 7, 14, 21, and 28 DIV and probed with a) TDP-43 antibody and b) Flag antibody with c) β-tubulin as loading control. Anti-Flag antibody showed the presence of the transgene-specific 43 kDa band (similar to TDP-43 band) only in mutants and not in control motor neuron lysates. d)Quantification of total TDP-43 (% normalized TDP-43) and transgenic TDP-43 (% normalized Flag) relative to β-tubulin over time reveals a decrease in total TDP-43 expressed by 28 DIV. It also indicates that there is no overexpression of the transgenic protein as the percentage of total TDP-43 in the mutant line is always less than the control cell line. *Blots were stripped and re-probed for each antibody.
